# Health and Fecundity of the Region around Fort Kent, Maine

**Published:** 1844-12

**Authors:** 


*Health and Fecundity of the Region around Fort Kent, Maine.*—These two highly interesting questions, both as regards the United States troops and the French Acadian population, are clearly illustrated by Dr. Wotherspoon, in the following extract from his letter, dated July 1:

"During the last two months not one of the men has been confined to bed by sickness, even for a single day, and the same universal good health seems to prevail in the surrounding country. One is the more inclined to wonder at this, on seeing the crowds of little unvaccinated wretches that swarm around the door of every log hut, half clothed, and but poorly fed on sour black bread and potatoes. Nothing has astonished me so much as the large families that are found among the French inhabitants. Five adjoining married couples have had in all 40 children, of whom, 35 are living. A man immediately opposite, on the other side of the St. John, has 27 by two wives, the second of whom bore 14. Lareut Ferriaud, at Green River, has had 25 children by one wife—no abortions. Buonaventure Le Crog had 19 children in 18 years; and of these, 5 pair were twins. Pierre Richou has had 6 in 3 years, 3 pair twins; all now living at Shattaquoi, six miles below the fort. Mr. Webber, the present State Commissioner, mentioned that in one house he found a woman with 5 children under 3½ years, one twin and one triplet birth. She was then a third time pregnant, her husband confidently expecting soon to be presented with another pair of the 'pretty prattlers.' I have had several females apply for advice for a prolapsus uteri and leucorrhœa, induced by too rapid child-bearing, and by the habit which all the women around here have, of arising from the bed the second or third day, in order to attend to their accustomed household duties. Day before yesterday, a pretty little French woman consulted me on this account; on inquiry, I found that she had had five children in six years. I think that 'Ould Ireland' can hardly hold a candle to the Madawaski plantation in either the production of children or potatoes."

This law of extraordinary fecundity and of equally remarkable health is not, however, in conformity to the law developed by statistics, which shows that the ratios of births and of deaths bear a direct relation.—*New York Journal of Medicine and the Collateral Sciences.*

